# Chlorpheniramine Potentiates the Analgesic Effect in Migraine of Usual Caffeine, Acetaminophen, and Acetylsalicylic Acid Combination

**DOI:** 10.3389/fphar.2017.00758

**Published:** 2017-10-24

**Authors:** Victor A. Voicu, Ion Mircioiu, Roxana Sandulovici, Constantin Mircioiu, Cristina Plesa, Bruno S. Velescu, Valentina Anuta

**Affiliations:** ^1^Department of Clinical Pharmacology, Toxicology and Psychopharmacology, Faculty of Medicine, “Carol Davila” University of Medicine and Pharmacy, Bucharest, Romania; ^2^Department of Biopharmacy and Pharmacokinetics, Titu Maiorescu University, Bucharest, Romania; ^3^Department of Applied Mathematics and Biostatistics, Titu Maiorescu University, Bucharest, Romania; ^4^Doctoral School, “Carol Davila” University of Medicine and Pharmacy, Bucharest, Romania; ^5^Department of Physiopathology, Titu Maiorescu University, Bucharest, Romania; ^6^Department of Pharmacology and Clinical Pharmacy, Faculty of Pharmacy, “Carol Davila” University of Medicine and Pharmacy, Bucharest, Romania; ^7^Department of Physical and Colloidal Chemistry, Faculty of Pharmacy, “Carol Davila” University of Medicine and Pharmacy, Bucharest, Romania

**Keywords:** migraine, analgesic synergism, pain survival curves, direct comparison of pain curves, Weibull

## Abstract

Previous studies indicated that addition of the antihistaminic chlorpheniramine to the usual combination of acetylsalicylic acid, acetaminophen, and caffeine further increases their synergism both in terms of anti-inflammatory and analgesic effect. The present non-interventional study tested the superiority of two Algopirin® tablets, containing a total of 250 mg acetylsalicylic acid (ASA), 150 mg acetaminophen (paracetamol, PAR), 30 mg caffeine (CAF) and 4 mg chlorpheniramine (CLF) vs. a combination containing 250 mg ASA, 250 mg PAR, and 65 mg CAF recognized as “safe and effective” by FDA in treating migraine. Patients evaluated their pain intensity on the Visual Analog Scale—VAS(PI) before and 30, 60, 120, 180, and 240 min after drug intake. Interpretation of the pain curves as “survival pain curves” was considered as a method for direct comparison of the pain curves. This interpretation permitted the application of the log rank test for comparison of pain hazards. The results of the applied parametric and non-parametric statistical tests indicated significant differences between the main endpoints: both Areas Under Pain Curves and time to decrease of the pain intensity to less than 50% of the initial value comparisons highlighted that Algopirin® was more efficient in spite of smaller doses of PAR and CAF. Comparison of “survival of pain” led to the same conclusion concerning the superiority of Algopririn. Consequently, the addition of CLF permitted decreasing of ASA, PAR, and CAF doses as well as their potential side effects, without a loss of analgesic effect.

## Introduction

Migraine is a high prevalence primary headache form, associated with a high socio-economic burden. The majority of people affected will treat their condition with medication. About 41% use prescription medications, either alone or in combination with over-the-counter (OTC) drugs (Lipton and Silberstein, 2015). The OTC medication used for migraine treatment includes single-ingredient formulations containing acetaminophen, acetylsalicylic acid or ibuprofen, as well as combinations containing no <250 mg acetaminophen (PAR), 250 mg acetylsalicylic acid (ASA), and 50 mg caffeine (CAF) per dosage form ASA-PAR-CAF (Lipton et al., 1994; Wenzel et al., 2003; Taylor and Smith, 2006; Reddy, 2013). In 1993, the Non-prescription Drug Advisory Board of the Food and Drug Administration recommended the classification of caffeine when combined with ASA and PAR as a category 1 analgesic adjuvant—“recognized as safe and effective” (Hersh et al., 2000). Caffeine shifts the dose–response curve to the left with an increase of analgesic potency of about 40% (Laska et al., 1984). This combination was classified by American Academy of Neurology as first-line migraine treatment (Silberstein, 2000).

The use of antihistaminergic substances in the pain management is sustained by some preclinical studies that stated that H1-receptor antagonists (benzhydramine mepyramine) potentiated analgesic action of morphine and fentanyl (Malec, 1987). Clorpheniramine (CLF), as a representative substance for the antiallergic antihistaminergic class does not have analgesic (Rumore and Schlichting, 1986; Raffa, 2001).

Although CLF does not have antiinflammatory or analgesic effects, our studies highlighted, using the rat paw model and a clinical study a supplementary potentiation of the both anti-inflammatory (Voicu et al., 2016) and analgesic effect (Blendea et al., 2011) of the overall combination following its addition to ASA-PAR-CAF.

A clinical study (Blendea et al., 2011; Enache et al., 2012) highlighted the non-inferiority of a unique dose of Algopirin®, product based on a new analgesic combination (Voicu et al., 2016) containing 125 mg ASA + 75 mg PAR + 15 mg CAF + 2 mg CLF vs. Excedrin®, a fixed combination drug containing 250 mg ASA + 250 mg PAR + 65 mg CAF. The effect was installed some 10–15 min more rapid in case of Excedrin® but the extent was approximately similar for the two combinations.

Taking into account that doses of active components in Algopirin® tablets are half of the doses in Excedrin®, from a global, efficacy and safety point of view, Algopirin® was considered as an alternative at least in case of patients with gastric and hepatic sensibility.

In the present study, the superiority of the treatment with two tablets of Algopirin® vs. one tablet of Excedrin® in the treatment of migraine was tested.

## Materials and methods

### Patients

The study was conducted in the Ilfov County Hospital, Romania. The study conformed with the Helsinki Declaration of 1964, as revised in 2013, and the protocol was approved by the “Carol Davila” University of Medicine and Pharmacy, Bucharest, Romania, Ethics Committee (approval number 31/15.12.2009) The study was conducted according to International Conference on Harmonization (ICH) Good Clinical Practices, to the Guidelines for Controlled Trials of Drugs in Migraine (Tfelt-Hansen et al., 2000) and to the Guidance on Clinical Investigation of Medicinal Products for the Treatment of Migraine (EMA., 2007).

All participants gave written informed consent prior to study participation and were instructed how to record the characteristics of their headache.

Male and female subjects, aged between 18 and 65 years (*n* = 46, 12 males and 34 females), were recruited by general practitioners or internal medicine specialists at the clinical facility. Diagnosis of headache was based on a structured questionnaire. The enrolled subjects fulfilled the criteria of the International Headache Society for episodic tension headaches and/or migraines with or without aura (Headache Classification Committee of the International Headache Society (IHS)., 2004). Patients were eligible for inclusion if they had experienced headaches for at least 12 months, with at least two episodes within the last 3 months. At least moderate pain intensity (with a score of at least 30 on the 1–100 units Visual Analog Scale) was also required.

Patients were excluded if they used prescription analgesics or antimigraine drugs, if they needed more than one dose of non-prescription analgesic to treat headaches or if they were under ongoing treatment with aspirin (over 100 mg/day), acetaminophen or caffeine containing drugs as well any other prescription or non-prescription analgesics. Patients receiving treatment with antidepressants or antipsychotics 1 month prior to the study, patients receiving anti-rheumatic or anti-inflammatory drugs in the last 4 days as well as the ones under ongoing treatment with anticoagulants were also excluded. Other exclusion criteria also included special physiological conditions (pregnancy, breastfeeding, female patients with period associated migraines), alcohol or drug abuse, hypersensitivity to ASA, acetaminophen or caffeine, different diseases (gastrointestinal ulcer, bleeding diathesis, glucose 6-phosphate dehydrogenase deficiency, asthma, liver disease, preexistent kidney disease, Gilbert syndrome or hyperthyroidism, major neurological disorders).

Headache lasting over 10 days/month or headache that left untreated lasts over 4 h as well as prolonged (over 10 days/month) analgesic treatment were also considered exclusion criteria.

### Study medication

Excedrin® (Novartis Consumer Health) was purchased from a community pharmacy, whereas Algopirin® was provided by LaborMed Pharma, Romania.

During the first 4 h after study medication, no other drug intake was allowed. Rescue medication was allowed only 4 h after the administration of the study medication. Two hours before and after intake of the study medication, the patients were not allowed to drink coffee or caffeine containing beverages.

### Study design

The study was designed as an open-label trial, extension of a randomized, two-sequences, two periods double-blind cross-over non-inferiority study previously described (Blendea et al., 2011), which compared one Algopirin® and one Excedrin® tablet.

Patients who received in the previous study one tablet of Excedrin® (containing 250 mg ASA, 250 mg PAR and 65 mg CAF) were called at the clinical site in case of a new migraine episode and received two Algopirin® tablets, containing a total of 250 mg ASA, 150 mg PAR, 30 mg CAF and 4 mg CLF.

From the 46 patients who received one tablet of Excedrin® in the non-inferiority study, 24 came to the extension phase, receiving two tablets of Algopirin®.

### Efficacy measurement

Pain Intensity was measured on a horizontal 100-mm Visual Analog Pain Intensity Scale [VAS(PI)] scale labeled: No Pain (0 mm) as the left anchor and Worst Pain Imaginable (100 mm) as the right anchor. During the screening visit, the investigator gave to all study participants a standardized explanation on how to record the VAS (PI) score, using a written explanatory text.

Patients were required to record in a headache diary the date and time of drug administration, baseline (immediately before treatment) pain intensity on the VAS (PI) scale, and pain intensity after treatment recorded at 30, 60, 120, 180, and 240 min, if the pain persisted 4 h after the administration of the study medication, if rescue medication was used (the drug and dose).

Clinically, with no absolute reference standard for pain measurement, individuals vary in the subjective rating of the pain intensity they indicate on the VAS (PI) scale. Thus, the actual value of the VAS (PI) score has no intrinsic meaning from a clinical perspective. Two different approaches were used in order to account for the differences in baseline pain intensity among patients. The first one was to normalize VAS (PI) scores, by expressing the values at intermediate time points as percent of the pain score taken at the predose baseline (considered as 100%). The second method, more common in literature (Hawker et al., 2011) was to convert the pain scores for each patient into Pain Intensity Difference (PID) scores, by subtracting them from the baseline pain score.

### Endpoints

Quantification of clinical effects is a difficult problem for all clinical studies, results and final conclusions being strongly dependent on the chosen markers of clinical effects as well as of their quantification. In the particular case of migraine the first marker is pain, its characteristics and time course giving possibility to compare different treatments.

Whatever the used scale and method, the final result is a number characterizing the pain intensity. Since pain is not limited to a single time point but to time intervals, and since its intensity is not constant over time, the evaluation at a series of time points leads to a “pain curve.”

The International Headache Society guideline for evaluating migraine therapy in clinical trials recommends evaluation of headache response 2 h after drug administration (Tfelt-Hansen et al., 2000). The guideline further recommends using the number of attacks resolved within 2 h as a primary endpoint, which is clearly unrealistic. Although this expectation is not usually met, this shorter time frame was chosen in the guideline in order to allow patients to take rescue medications after 2 h.

European Medicines Agency (EMA., 2007) recommends as possible secondary endpoints the percentage of patients remaining pain-free (defined as being pain-free at 2 h with no use of rescue medication and no relapse within 48 h after administration of the study agent), the intensity of headache at various time points etc.

#### Primary endpoint

The primary endpoint of the trial was time to 50% pain relief (T_50_). The parameter was estimated as time corresponding to intersection of the 50% gridline of pain intensity curves, and its value was calculated for each subject by linear interpolation between adjacent observation time points.

#### Secondary endpoints

As secondary endpoints, time until reduction of pain intensity to 20% (Time to 80% pain relief -T_20_) and to 10% (Time to 90% pain relief -T_10_) on the VAS(PI) scale, as well as the percentage of patients with at least 50% pain relief after 1 h and percentage of patients pain free 4 h after treatment were considered.

#### Direct comparison analysis of “pain curves”

Additionally, for a more in depth analysis some methods for direct comparison of “pain curves” were considered.

Area under Curve (AUC) is a natural global parameter useful in comparison of curves. For instance, area under plasma levels curves of active substances is the most significant parameter in defining bioavailability of a drug. Different statistical methods are used in order to assess AUC, of which the most common is the trapezoid rule:

$$\begin{array}{l} AUC=\overset{n}{\underset{i=1}{\sum }}\frac{f\left(t_{i-1}\right)+f\left(t_{i}\right)}{2}*\left(t_{i}-t_{i-1}\right) \end{array}$$

where *f*(*t*_*i*−1_) and *f*(*t*_*i*_) correspond to consecutive measurement time points. For the present study, by assimilating the time course of pain score with a “pain curve,” the Area Under Pain Curve (AUPC) can be evaluated and used as comparison tool.

AUPC was calculated using a similar formula:

$$\begin{array}{lll} AUPC_{0\to t} & = & \sum AUPC_{t_{i}-1\to t_{i}}\\ =\frac{\sum \left[PI\left(t_{i-1}\right)+PI\left(t_{i}\right)\right]*\left(t_{i}-t_{i-1}\right)}{2}, \end{array}$$

where PI(*t*_*i*_) and PI(*t*_*i*−1_) correspond to two pain intensity measurements at consecutive time points, expressed as normalized VAS(PI) scores.

The values for AUPC form 0 to 2 h and from 0 to 4 h were all considered endpoints in the present study.

Similarly, considering the PID curve, derived by subtracting the pain score at each post-dosing time point from the baseline score, a parameter somewhat similar with AUPC, called Sum of PID differences (SPID) can be obtained (Blendea et al., 2011). In fact SPID is the sum of PID scores multiplied by the interval between ratings:

$$\begin{array}{l} SPID=\overset{n}{\underset{i=1}{\sum }}PID\left(t_{i}\right)\left(t_{i}-t_{i-1}\right) \end{array}$$

For slow decreasing curves the areas are approximately equal to SPID but for time intervals with rapid change of pain, the differences can no more be neglected.

Additionally, Kaplan-Meier Survival Analysis (Kaplan and Meier, 1958) was performed in order to evaluate the distribution of the time to total pain relief, and to and compare the effectiveness of the two treatments. Comparison of mean pain curves was performed using Cox-Mantel test, also called log-rank test (Peto and Peto, 1972).

### Statistical analysis

The statistical analyses as well as graphical representation of data were performed using GraphPad Prism 7 (GraphPad Software Inc., La Jolla, CA, United States) software.

For continuous variables, descriptive statistics (mean, standard deviation, frequencies) were calculated. In order to apply the parametrical tests, the normal distribution of the results was verified both visual and using D'Agostino-Pearson normality test.

Statistical comparisons of different numerical data sets (demographic data such as age, weight, BMI, as well as study endpoints such as T_50_, T_20_, T_10_, AUPC_0−2h_, AUPC_0−4h_, SPID_0−2h_, SPID_0−4h_) were performed using Student's T-test. When only testing the superiority hypothesis one-tailed T-test was applied, whereas for general comparison purposes two-tailed T-test was used. The results were considered statistically significant when *P*-values were < 0.05.

Comparisons using Chi-square test were performed (significance level-*P* < 0.05) for categorical variables.

Survival curves were obtained using the Kaplan-Meier method. Differences between the resulted survival curves were assessed according to the log rank test. Differences were considered significant at values of *P* < 0.05.

In terms of evaluationg the apropriateness of statistical test applied, in a recent paper we underlined that “a trenchant and passionate dispute over the use of parametric vs. non-parametric methods in clinical data has raged in the literature for the past eight decades” (Mircioiu and Atkinson, 2017).

Our conclusion was that answer is not a simple “yes” or “no” but is related to hypotheses, objectives, risks, and paradigms. In a pragmatic approach, we argued that a better way is to apply both type of tests, to compare results and finally to apply clinical criteria in evaluating the likelihood of results.

Usually, in clinical trials concerning analgesia sample size are estimated in order to ensure 0.8 power to detect differences between treatments, considering the significant difference in clinical success rate Δ = 20% based on VAS(PI) evaluation (Diener et al., 1999). Considering the probability of type I error α = 0.10 for type II error β = 0.20, a coefficient of variation ($CV=\frac{\sigma }{\mu }*100$) of 40 %, and a normal distribution of the area under the pain curves, a necessary number *n* = 71 subjects was obtained.

## Results

### Characteristics of the study groups

Details on the demographic characteristics of patients enrolled in the study are presented in Table 1. No significant differences were found between the main demographic characteristics: age (T-test, *P* = 0.5303), gender repartition (Chi-square test, *P* = 0.9213), weight (T-test, *P* = 0.8406), body mass index (T-test, *P* = 0.5666), as well as clinical aspects: number of migraine episodes in the last month (T-test, *P* = 0.9013), and mean pain intensity of the untreated migraine expressed as VAS(PI) score (T-test, *P* = 0.6705).

**Table 1:** Demographics and baseline pain characterisctics of the two treatment groups.

**Parameter**	**Excedrin® group**	**Algopirin® group**	***P-*value**
No. of subjects (n)	46	24	–
Male/Female (n/n)	12/34	6/18	0.9213 (ns)
Age (years)	36.35 ± 9.24	38.29 ± 8.96	0.5303 (ns)
Weight (kg)	68.35 ± 12.99	69.12 ± 14.6	0.8406 (ns)
Body Mass Index (kg/m^2^)	23.96 ± 4.126	24.69 ± 5.26	0.5666 (ns)
No. of migraine episodes in the last month	3.40 ± 2.06	3.29 ± 1.80	0.9013 (ns)
Mean pain intensity of the untreated migraine	64.78 ± 19.29	62.35 ± 21.95	0.6705 (ns)

*ns, not significant*.

The demography of the study population included into clinical trials should mimic that of the patient group to whom it will eventually be prescribed, young and middle-aged women were recruited predominantly in the study, since migraine has higher frequency in this population group (Goldstein and Chen, 1982). The male to female ratio in the Excedrin® and Algopirin® patient groups was 1:2.8 and 1:3 respectively, with no significant difference in gender distribution between the two groups (Chi-square test, *P* > 0.5) (Table 1).

### Efficacy results

#### Visual inspection of the clusters of individual curves

**Figure 1:**
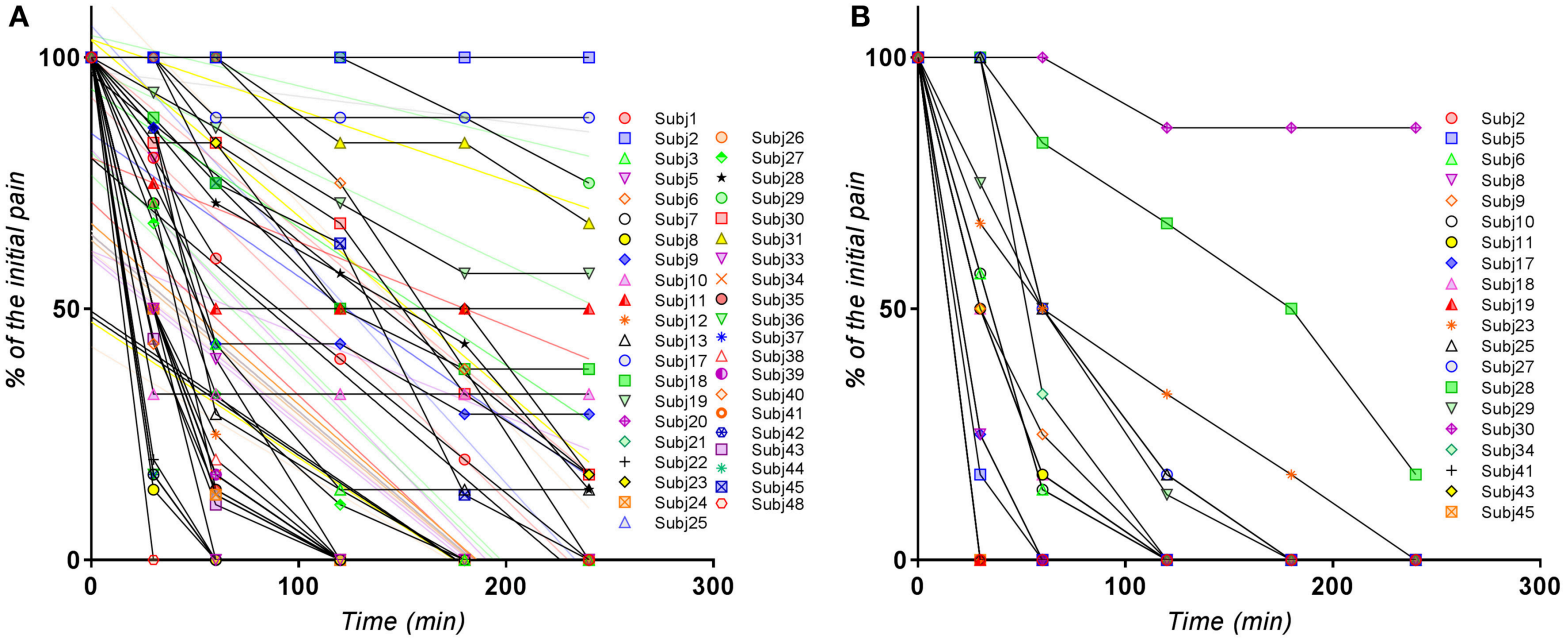
Individual normalized VAS(PI) score values for **(A)** Excedrin® treatment, **(B)** Algopirin® treatment.

The visual analysis of the entire set of individual curves (Figure 1) allowed the identification of the outlier curves (as for example in case of Excedrin® a subject with zero response). It appeared that the curves are more or less homogeneously distributed and not divided into subclusters (for example of poor and extensive metabolism).

The global evaluation of the clusters of pain curves revealed a shifting toward lower VAS(PI) score values in case of the Algopirin® treatment in comparison with the Excedrin® treatment. In the Excedrin® cluster most of the pain curves are placed in the middle and lower region of the graph, whereas in the Algopirin® cluster the highest density of curves is in the lower part of the scale. Therefore, at a direct visual analysis, it appears that Algopirin® is more effective in reducing pain.

#### Mean curves

**Figure 2:**
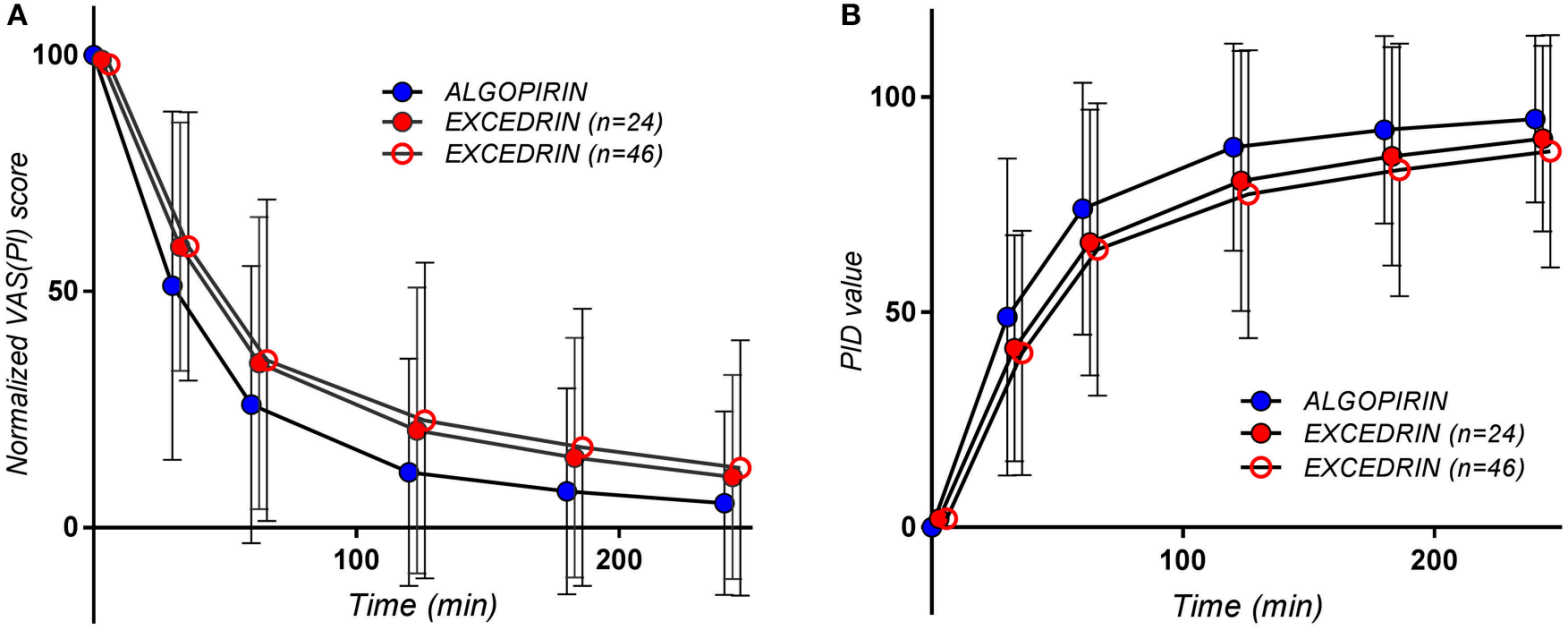
The pain curves (Mean ± *SD*) obtained for the patients treated with Algopirin (*n* = 24; blue circles), for all the patients treated with Excedrin (*n* = 46, empty red circles) and for Excedrin only in case of patients participating in the present study (*n* = 24, full red circles), expressed as **(A)** normalized VAS(PI) score values vs. time and **(B)** PID values vs. time.

The mean pain curves for Algopirin®, and Excedrin® respectively, calculated based on normalized individual VAS(PI) data are depicted in Figure 2A. Since it is more intuitive to associate better efficacy with higher numerical values, a representation based on the mean of Pain Intensity Differences (PID), calculated as the complement of VAS(PI) pain intensity (100–value), was also used (Figure 2B).

### Estimation of the parameters associated with the mean and individual pain curves

#### Time to a fixed % pain relief

For the Excedrin® set, T_50_ distribution clearly appears bimodal, phenomenon absent in the case of Algopirin®. A possible explanation for the obtained could be the occurrence of some allergic reactions after Excedrin® treatment, which were countered by the clorpheniramine component of Algopirin®.

**Figure 3:**
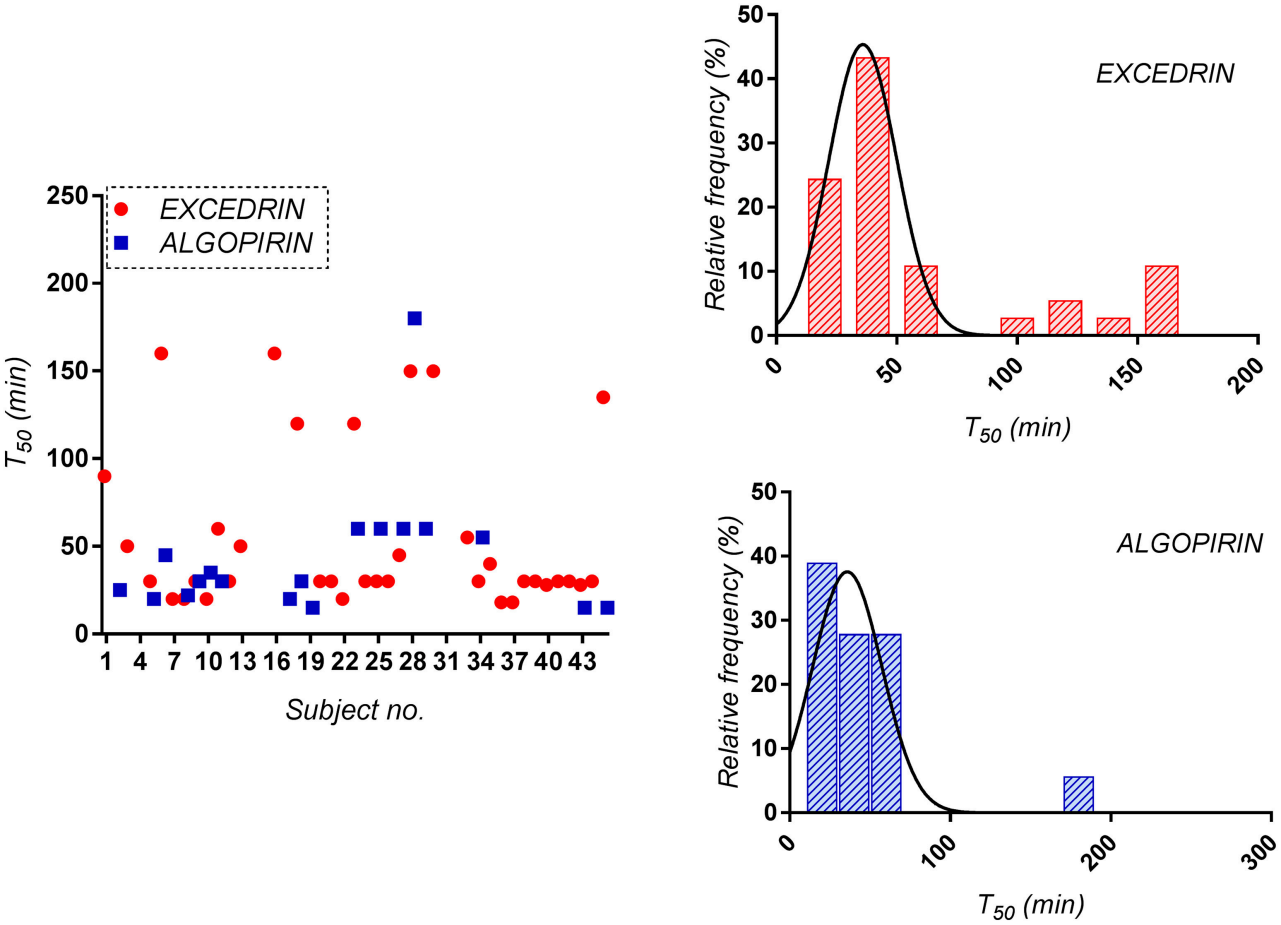
Distribution of the time to 50% pain relief (T_50_) for both Algopirin® and Excedrin® individual pain curves.

For mean curves T_50_ was 30 min for Algopirin® and 45 min for Excedrin® (Figure 3). The 15 min difference between the two treatments can be considered somewhere above the limit between clinical significant and non-significant difference.

Time to 90% pain relief (T_10_) was calculated by linear interpolation or extrapolation and the mean experimental values were found to be 180 min for Algopirin® and 240 min for Excedrin® respectively.

**Figure 4:**
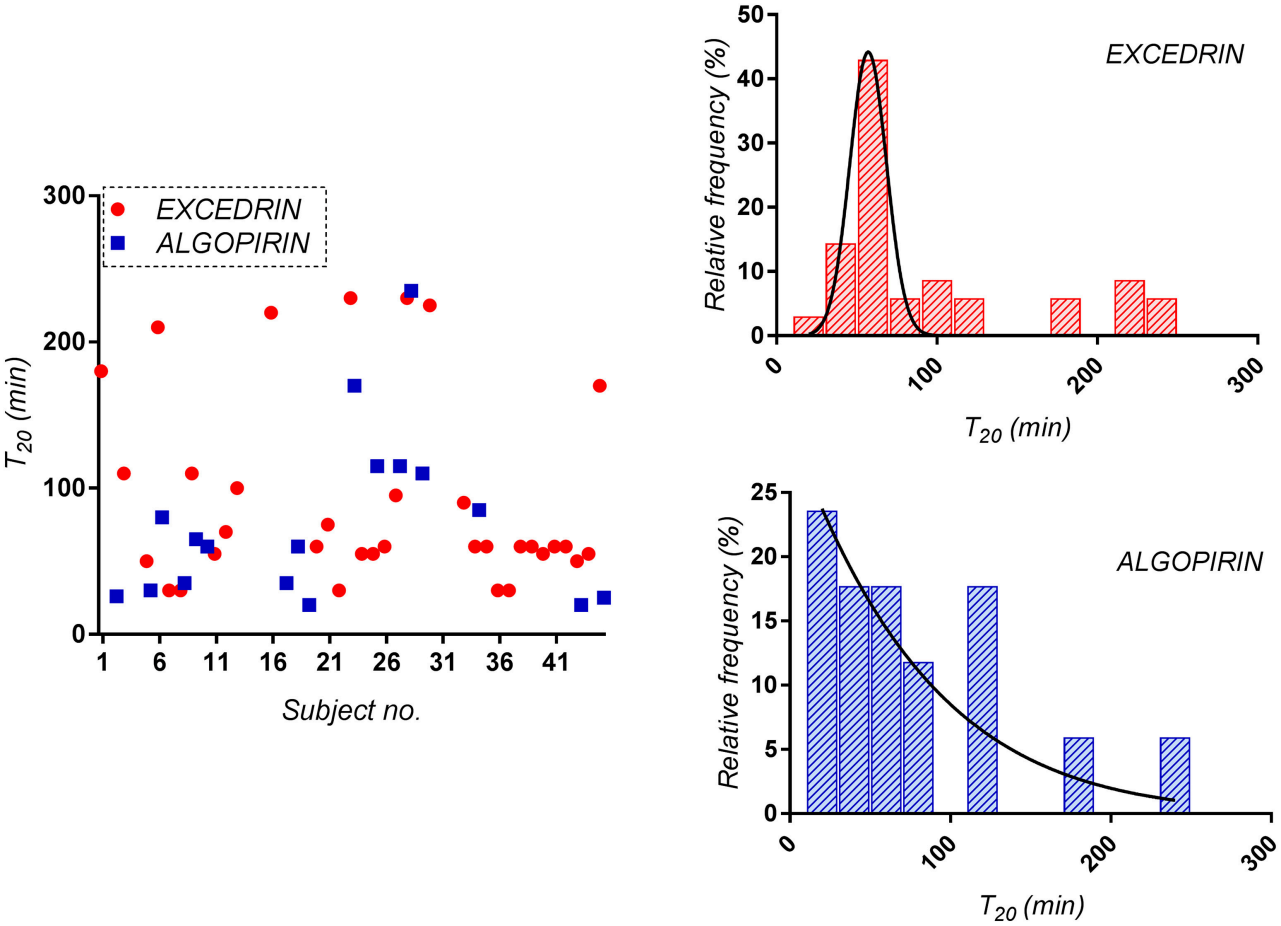
Distribution of the time to 80% pain relief (T_20_) for both Algopirin® and Excedrin® treatments.

Considering as significant clinical difference at least 20% difference in effect, time to 80% pain relief (T_20_) was considered as alternative to T_10_. In the present study, T_20_ was 80 min for Algopirin® and 130 min for Excedrin®, suggesting that Algopirin® is more efficient than Excedrin® (Figure 4).

**Figure 5:**
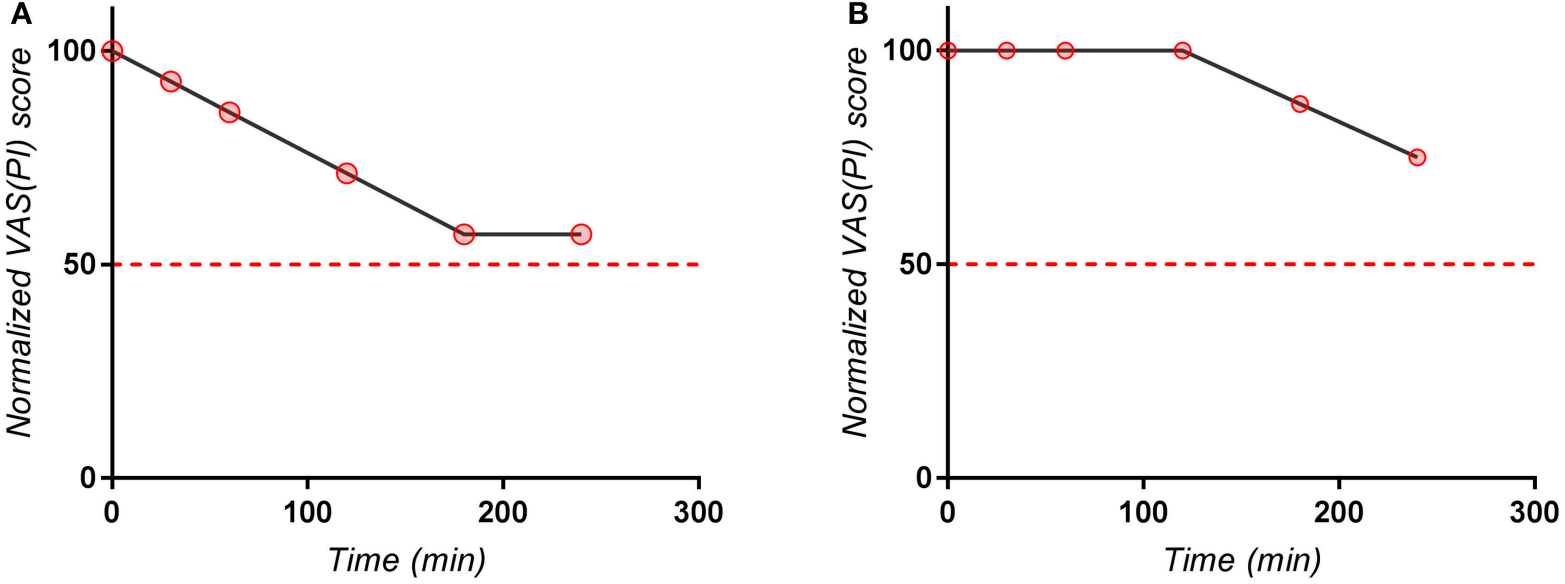
Individual normalized VAS(PI) score values for **(A)** a subject with an “outlier” last point, **(B)** Subject with a lag in apparition of effect.

Evaluation of T_50_ for each patient is useful for statistical analysis, but many difficulties may occur in practice, due to some particular more or less outlier curves. For instance, one subject (Figure 5A) had the last recorded point for Excedrin® above the 50 score line. In this case, the curve didn't intersected the 50 score line, but the curve associated with majority of experimental data points (from 0 to 180 min) was linearly extrapolated and 210 min was determined as T_50_. In case of another subject (Figure 5B) it seems that T_50_ for Excedrin® is around 5–6 h, which is beyond the recorded time. In this case, 240 min was considered as truncated T_50_.

Evaluation of individual data for detection and elimination of outlier data or curves was until recently considered practically unacceptable by drug authorities the Food and Drug Administration on Statistical Approaches to Establishing Bioequivalence stating that “deletion of outlier values is generally discouraged” (FDA., 2001).

However, sometimes a single outlier is sufficient to overthrow all results. For instance, inclusion of the patients without pain relief into calculations would completely change the final result, as it would lead to an infinite mean value for T_50_. On the other hand, calculation of T_50_ based solely on the mean curve would hide all outliers and the result could be considerably biased.

Introduction of scaled criteria in estimation of bioequivalence of drugs led to the observation that outliers in case of reference drugs lead to increase of variance and artificial increase of the length of acceptance intervals which fact changed the opinion of FDA on outliers treatment and analysis (FDA., 2016).

Two different types of comparisons between treatments were performed: one comparison between all the patients who received Excedrin® (*n* = 46) and patients receiving Algopirin® (*n* = 24), and the other one taking into consideration only the patients receiving both treatments (*n* = 24).

As can be seen in Tables 2, 3, comparisons of T_50_ and T_20_ parameter put in evidence a statistical significant difference between the values associated to the two treatments in both type of calculus.

**Table 2:** Comparison of the primary and secondary endpoints of the clinical trial, for patients receiving both Excedrin and Algopirin treatments.

**Parameter**	**Excedrin® group**	**Algopirin® group**	***P-*value**	**Significance**
T_50_ (min)	51.07 ± 40.3	30.14 ± 14.04	0.0191	^*^
T_20_ (min)	142.4 ± 142.4	76.47 ± 58.86	0.0024	^**^
AUPC_0−2h_	120 ± 58.50	64.13 ± 34.62	0.0019	^**^
AUPC_0−4h_	188.6 ± 118.90	77.58 ± 59.37	0.0010	^***^
Patients with at least 50% pain relief after 1 h (%)	55.6	88.9	0.0128	^*^
Patients pain free after 4 h (%)	67.39	91.30	0.0149	^*^

Levels of significance: ns, not significant (P > 0.05);

* P ≤ 0.05,

** P ≤ 0.01,

*** *P ≤ 0.001*.

**Table 3:** Comparison of the primary and secondary endpoints of the clinical trial for the entire population of patients receiving Excedrin (*n* = 46) and Algopirin (*n* = 24).

**Parameter**	**Excedrin® Entire group**	**Algopirin® group**	***P*-value**	**Significance**
T_50_	48.79 ± 41.22	30.14 ± 14.04	0.0122	^*^
T_20_	120.5 ± 84.48	76.47 ± 58.86	0.0152	^*^
AUPC_0−2h_	93.04 ± 54.05	64.13 ± 34.62	0.0094	^**^
AUPC_0−4h_	132.1 ± 109.6	77.58 ± 59.37	0.0082	^**^
Patients with at least 50% pain relief after 1 h (%)	74.36	88.9	0.0727	ns
Patients pain free after 4 h (%)	58.30	91.30	0.0048	^**^

Levels of significance: ns, not significant (P > 0.05);

* P ≤ 0.05,

** P ≤ 0.01, ^***^ P ≤ 0.001.

#### Area under pain curves (AUPC)

Considering that the effect depends on the concentration in blood of active substances, AUPC might be considered a more adequate parameter compared to usual parameter Sum of Pain Intensity Differences (SPID). Converting in percentages of the maximum possible value (number of hours^*^ 100, if the effect is absent) AUPC values at 2 h, respectively at 4 h were calculated (Tables 2, 3).

It can be seen that that AUPC for Excedrin® are greater than the ones for Algopirin® (Figure 2A), differences being statistically significant (Tables 2, 3) in both types of comparisons.

Since it is common to discuss about time to total pain relief (TOTPAR), an extrapolation of the pain intensity curves in order to compute the total AUPC (*AUPC*_0−∞_) was attempted. A technical problem in calculating this parameter is the calculation of the extrapolated area from the last measured point to infinity. This extrapolation is common in pharmacokinetics, based on the observation that, on the terminal elimination phase data points follow an exponential decay. Applicability of this method for the present study can be verified simply by logarithmic scale representation of the pain score data. If the terminal phase data can be fitted by a linear regression with slope k, monoexponential decay can be considered, and the extrapolated area will consequently be:

$$\begin{array}{l} AUPC_{t_{n}-\infty }=\int_{t_{n}}^{\infty }e^{-kt}dt=\frac{0-e^{-kt_{n}}}{-k}=\frac{PI\left(t_{n}\right)}{k} \end{array}$$

**Figure 6:**
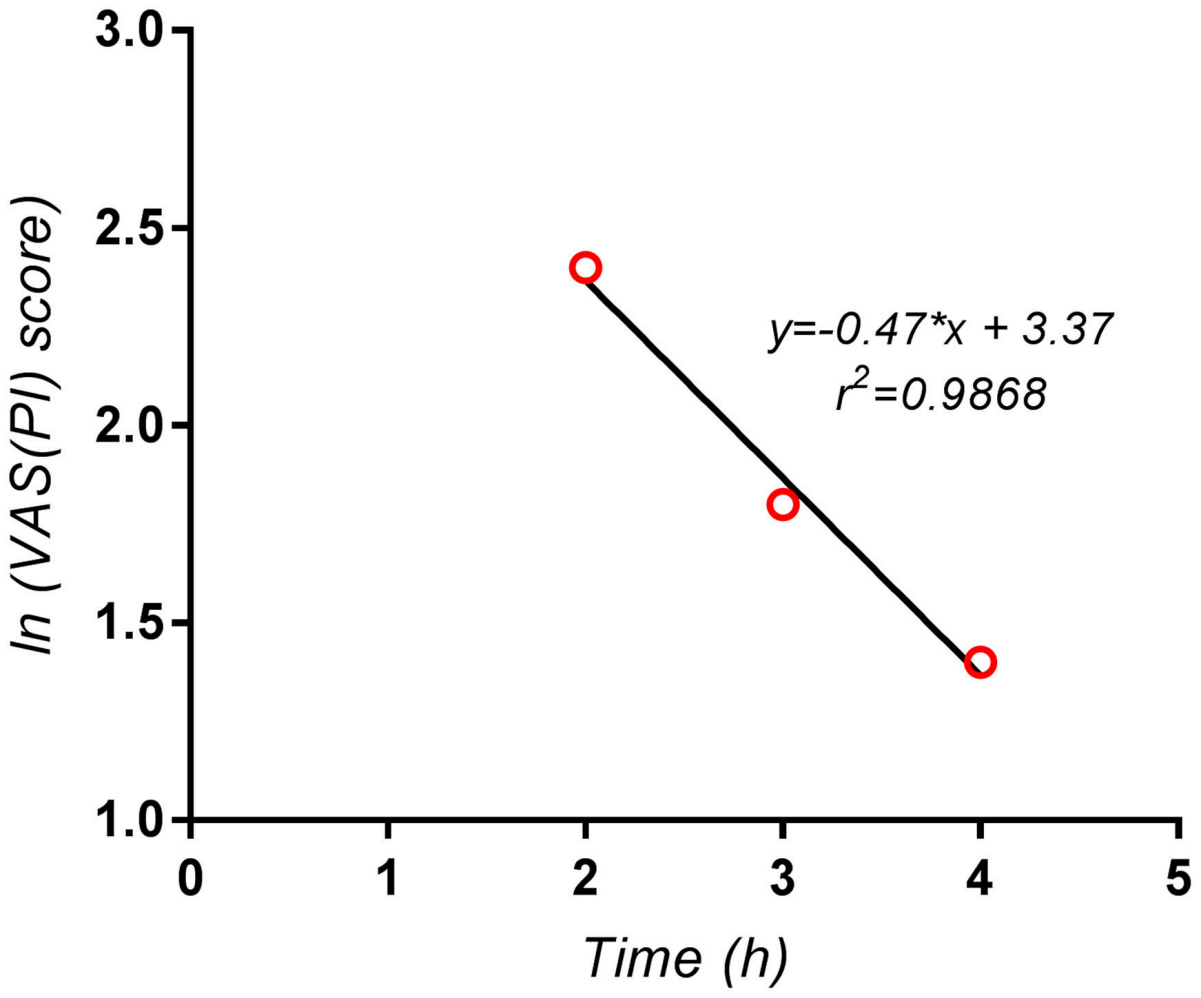
Logarithmic scale representation of the terminal phase (2–4 h) of the pain curve for the mean Algopirin® curve.

Since a exponential decay of the terminal phase of the pain curves could could be established in the present study, as can be seen for example in case of the Algopirin® mean curve (Figure 6), the above method was used in order to evaluate *AUPC*_4*h*−∞_ and *AUPC*_0−∞_ respectively.

The extrapolated area *AUPC*_4*h*−∞_ was in this case $\frac{4}{0.47}=8.5\;$VAS(PI) score^*^hours, representing <3% of *AUPC*_0−4*h*_. Consequently, the use of the extrapolated area instead of *AUPC*_0−4*h*_ had no significant impact on the test results, and did not influence the conclusions concerning comparison of the two treatments.

### Direct comparison of the pain curves (see also the supplementary material)

#### Cox-mantel method

Each individual pain curve can be reduced to a series of points, corresponding to the VAS(PI) scores measured at each of the measuring times. In the timeframe between two consecutive measuring times, some of the points may disappear, following the effect of drug or a physiological evolution independent of drug. Therefore, it is possible to associate the disappearance of some points due to the two above mechanisms (i.e., pain disappearance) and death of the patients in the classical survival analysis. Hence, it is suitable to treat the pain curves by means of the survival analysis, and to apply the Cox-Mantel method for comparison of means of “pain survival curves” and, if differences are the same at all measuring times the sum $X_{\log rank}=X_{LR}=\frac{{\left(\sum_{i=1}^{k}d_{1i}-\sum_{i=1}^{k}e_{1i}\right)}^{2}}{\sum_{i=1}^{k}D\left(d_{1i}\right)}=\frac{{\left(d_{1}-e_{1}\right)}^{2}}{\nu_{1}}$ is distributed χ^2^(α, 1).

For the explanation of the symbols used see also the Supplementary Material.

An estimation of the ratios of hazard functions and the confidence interval for it will be: $\hat{\lambda }=\exp\left(\frac{d_{1}-e_{1}}{v_{1}}\right)$ and $\exp\left\{\left[\left(d_{1}-e_{1}\right)/v_{1}\right]\pm Z\left(\alpha /2\right)/\sqrt{v_{1}}\right\}$ respectively.

**Figure 7:**
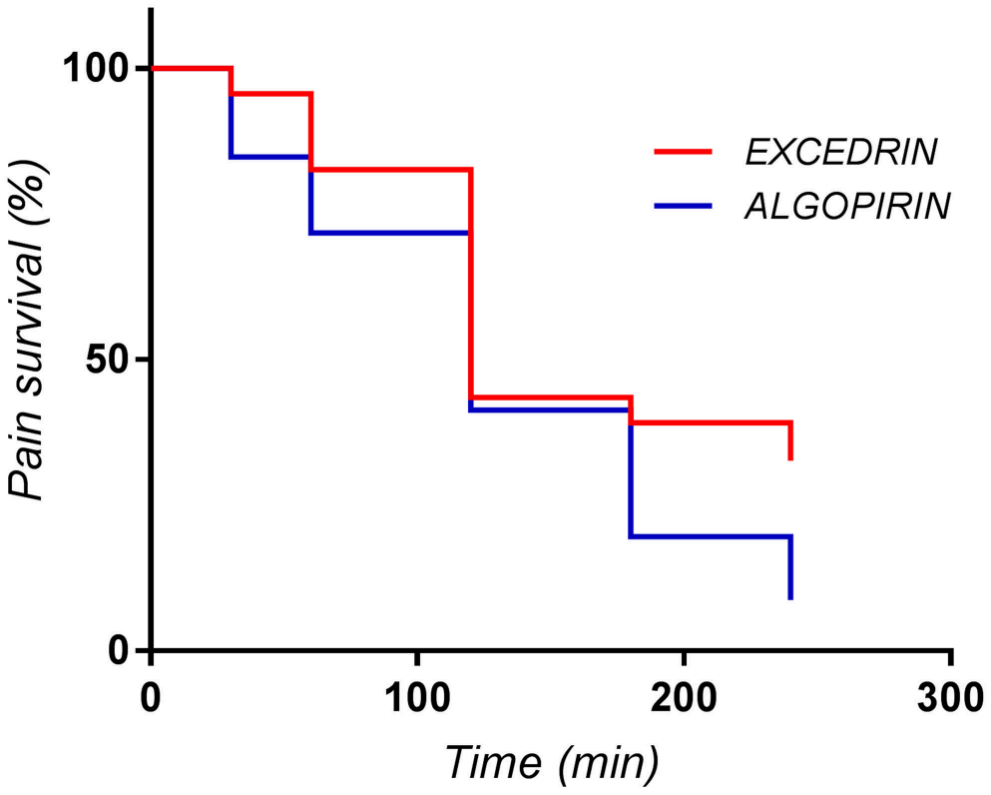
Mean “pain survival curves” for the Excedrin® (*n* = 46) and Algopirin® (*n* = 24) treatment.

The results for the present study are presented in Figure 7, Table 4 respectively.

**Table 4:** Estimation and comparison of Algopirin® and Excedrin® “pain survival curves.”

***t***	***d_1*k*_***	***d_2*k*_***	***d_*k*_***	***n_1*k*_***	***n_2*k*_***	***n_*k*_***	***e_1*k*_***	***d_1*k*_-e_1*k*_***	***ν_1*k*_***
0	0	0	0	100	100	200.00	0.00	0.00	0.00
30	49	39	87	51	61	112.60	39.69	9.18	4.89
60	25	24	49	26	37	63.36	20.22	4.90	2.70
120	14	13	27	12	25	36.20	8.69	5.75	1.52
180	4	6	10	8	19	26.53	2.78	1.18	1.31
240	3	4	7	5	15	19.70	1.78	0.72	0.90
**Sum**	**94.88**	**85.42**	**180.30**				**73.14**	**21.74**	**11.32**

X_*LR*_ = 41.74 $\hat{\lambda }$ = 6.82 CI 90%: (3.05;15.25).

where $e_{1k}=\frac{n_{1k}d_{k}}{n_{k}}$ and $\nu_{1k}=\frac{n_{1k}n_{2k}d_{k}\left(n_{k}-d_{k}\right)}{n_{k}^{2}\left(n_{k}-1\right)}$.

Since decision threshold is χ^2^(α, 1) = χ^2^(0, 90, 1) = 2, 71 and the obtained value is greater than the threshold, the conclusion is that the pain curves are significantly different and the analgesic effect of two Algopirin® tablets is superior to the effect of one Excedrin® tablet (*p* < 0.001).

#### Weibull model

Weibull model is one of the most general model in science, being applicable to processes running in several steps with constant rate of transfer (Weibull, 1951). In case of survival curves, this particularly means that the instantaneous death rate is constant at all measuring times. In our case, “number of pain points” defined above–n_ik_, could be described by Weibull distribution in the form:

$$\ln(-\ln(1-n_{ik}(t)/100)=\ln\alpha +\beta \text{​}\ln\text{​}t$$

Consequently, if ln (−ln (1−*n*_*ik*_(*t*)/100)vs. ln t describes a linear dependency, we can conclude that Weibull model is applicable.

**Figure 8:**
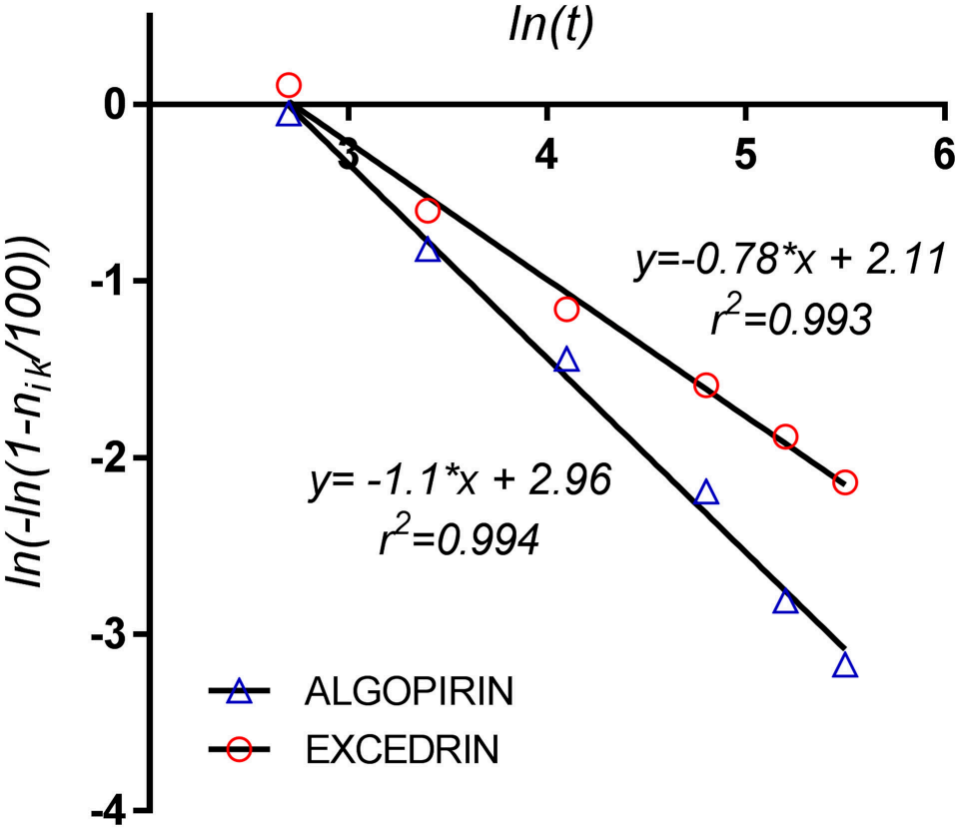
Weibull model for the pain survival curves.

The excellent linear fitting of the experimental data (Figure 8) prove that the application of the model is appropriate, which can suggest that the active components have the same action mechanism during the first 4 h after administration. Following the Cox proportional hazard model, if Weibull model applies to both curves (Algopirin® and Excedrin®) the obtained lines have to be parallel.

Explanation of the fact that the regression lines are not parallel (Figure 8) could be that mechanism of interaction between CLF and ASA-PAR-CAF combination is more complex than a simple addition.

## Discussion

In fact, the envisaged combination act on multiple mediators of the inflammatory process, including prostaglandins, histamine and therefore on the vascular permeability and cell membranes. Due to their action by different mechanisms—local analgesia (the anti-inflammatory mechanism by inhibition of prostaglandin synthesis by ASA); central analgesia (PAR); local antihistaminic and decongestant effect (the antihistaminic component); decreased vascular permeability and increased peripheral vascular tone, both leading to inflammation decrease through central mechanism (CAF)—the composition acts at different functional levels and through different ways. This complex action represents the complex pharmacodynamic background of the four associated components potentiating synergism.

In other words, while the literature presents mostly cases of synergism by addition (where the final results are at best the sum of the effects of the components), our compositions present a pharmacodynamic potentiation synergism, where the sum of the combined results is higher than the sum of the results of each ingredient taken individually. This is possible following the action of each ingredient through different mechanisms and different targets, allowing the incentive obtained result, that is: the smaller dose with the lower side effects, and better tolerability.

Different clinical trial data reports that the acetaminophen, acetylsalicylic acid and caffeine combinations are generally well-tolerated (Lipton et al., 1998; Goldstein et al., 2006). However, most of the reports relate to short-term administration whereas the long term use can lead to various serious adverse effects, such as gastrointestinal perforation (Lanas et al., 1997), agranulocytosis, plasmacytosis, and thrombocytosis (Gursoy et al., 1996), kidney failiure (Perneger et al., 1994), or acetaminophen-induced hypotension (Brown, 1996). In the label of Excedrin are mentioned as adverse effects “GI upset/bleed, prolonged bleeding time, urticaria, anaphylaxis; overdosage: salicylism, hepatoxicity.” In this context, the new ASA-PAR-CAF-CLF association allows the use of lower doses and obtaining a superior effect, which can be a major advantage in terms of drug safety of long term treatments.

## Conclusions

Consideration of Areas Under Pain Curves (AUPC) is preferable to the usual SPID parameter, being more sensitive in detecting the global difference between pain curves. Supplementary, the concept permits an analogy with areas under plasma levels of active compounds which are responsible for the therapeutic effect.

Interpretation of pain curves as “survival pain curves” brings a new approach in the methods of statistical analysis of the pain phenomenon, allowing application of more sophisticated methods from cancer analysis. Since no pain comparison method can be considered a “gold standard”, these approaches, in conjunction with the conventional ones, offer a mosaic of complementary methods in quantification and comparison of therapeutic effects.

The results were practically similar whatever the applied statistical test: two tablets of Algopirin® were more efficient than one tablet of Excedrin®. Addition of CLF increased the analgesic effect of ASA-PAR-CAF combination. Therefore, Algopirin®, in spite of lower doses of ASA, PAR and CAF has a greater analgesic effect and potentially lower adverse effects than the ASA-PAR-CAF alone.

## Author contributions

VV was the Principal Investigator of the clinical trial and coordinated the entire Algopirin® project, IM, BV, VA, and CP performed pharmacological and clinical experiments, RS and CM performed the mathematical and statistical analysis of the data, CM and VA prepared the manuscript. All authors approved the manuscript.

### Conflict of interest statement

The authors declare that the research was conducted in the absence of any commercial or financial relationships that could be construed as a potential conflict of interest.
