# Supplementary material for: Chlorpheniramine Potentiates the Analgesic Effect in Migraine of Usual Caffeine, Acetaminophen, and Acetylsalicylic Acid Combination
Source: Front Pharmacol. 2017 Oct 24;8:758. doi: 10.3389/fphar.2017.00758 (PMC5660851; doi:10.3389/fphar.2017.00758)
Supplement: Supplementary file 1 [file DataSheet1.DOCX]

Supplementary Material

Chlorpheniramine Potentiate the Analgesic Effect in Headache of Usual Caffeine, Acetaminophen and Acetylsalicylic acid Combination

Victor Voicu^1^, Ion Mircioiu^2^, Roxana Sandulovici^3^, Constantin Mircioiu^4*^, Cristina Plesa^5^, Bruno Stefan Velescu^6^, Valentina Anuta^7^

*** Correspondence:** Constantin Mircioiu: constantin.mircioiu@yahoo.com

Appendix A: Kaplan Meier estimation of the survival curve

In survival analysis it is usually of interest to obtain statistical inference from initiation of treatment to the occurrence of a predefined event (for example disappearance or death of a patient.

The cumulative distribution function (cdf) of T_s_, denoted by F(t), is defined as the probability that a subject fails before or equal to the time t, The cumulative distribution function (cdf) of T_s_, denoted by F(t), is defined as the probability that a subject fails before or equal to the time t, $F\left( t \right)=P\left( a subject fails before or equal to t \right)=P\left( T_{s}\leq t \right), 0<t<\infty$.

The survival function is the probability that a subject survives longer than t, namely $S\left( t \right)=1-F\left( t \right)=P\left( T_{s}>t \right), 0<t<\infty$.

The hazard function, denoted as h(t), is the instantaneous death rate, which is the conditional probability that a subject fails over the next instant given that the subject has survived up to the beginning of the interval.

Let $t_{1}<\ldots<t_{k}$ be the ordered distinct times when the event occurs and $d_{k}$ the number of events at time $t_{k}$. The number of subjects exposed to risk at time $t_{k}$ consists of $n_{k}$ subjects who are still alive just before $t_{k}$ and whose survival time is not censored before $t_{k}$.

The Kaplan-Meier nonparametric estimation of the survival function at time t is given by the formula: $\hat{S}\left( t \right)=\prod_{t_{k}<t} \left( 1-\frac{d_{k}}{n_{k}} \right)$.

In practice, from reasons connected with modeling and estimation of variance, is used the hazard cumulative function defined as H(t) = −lnS

Function hazard h(t), denoted sometimes $\lambda(t)$ is defined as derivative of H:

$$h\left( t \right)=H^{'}\left( t \right)=\left( -lnS \right)^{'}=\frac{{-S}^{'}\left( t \right)}{S\left( t \right)}$$

Appendix B: Comparison between “survival functions of pain”

One commonly used method for comparing two survival (curves) functions is the log rank test. $H_{0}: S_{1}\left(t\right)=S_{2}\left(t\right)$ vs $H_{a}: S_{1}\left(t\right)=\left[S_{2}\left(t\right)\right]^{\lambda}$or, after logarithmic transformation $\log S_{1}\left(t\right)=\lambda\log\left[S_{2}\left(t\right)\right]$ i.e. logarithms of survival curves are proportional.

The hypergeometric distribution is a discrete [probability distribution](http://en.wikipedia.org/wiki/Probability_distribution) that describes the number of successes in a sequence of *n* draws from a finite [population](http://en.wikipedia.org/wiki/Population) *without* replacement, just as the [binomial distribution](http://en.wikipedia.org/wiki/Binomial_distribution) describes the number of successes for draws *with* replacement.

|  | drawn | Non- drawn | Total |
| --- | --- | --- | --- |
| white | $k$ | $m-k$ | $m$ |
| black | $n-k$ | $N+k-n-m$ | $N-m$ |
| Total | $n$ | $N-m$ | $N$ |

$$P\left(white\right)=\frac{m}{N}$$

The mean and variance of this distribution are:

$$E\left(X\right)=n*\frac{m}{N}$$

$$D\left(X\right)=n*\frac{m}{N}*\left(1-\frac{m}{N}\right)*\left(\frac{N-n}{N-1}\right)$$

If the n draws are made at once, then $D\left(k\right)=np\left(1-p\right)\frac{N-n}{n-1}$.

Using the usual notations in survival curves:

|  | Group 1 | Group 2 |  |
| --- | --- | --- | --- |
| failed | $d_{1i}$ | $d_{2i}$ | $d_{i}$ |
| alive | $n_{1i}-d_{1i}$ | $n_{2i}-d_{2i}$ | $n_{i}-d_{i}$ |
|  | $n_{1i}$ | $n_{2i}$ | $n_{i}$ |

If the tested hypothesis is the identity of the two survival curves, we can reunite the numbers of failed and survival patients at a given time $\left[\left(d_{1i} , n_{1i}\right) and \left(d_{2i} , n_{2i}\right)\right]$in order to estimate common distribution. Proportion of failed will be $\frac{d_{i}}{n_{i}}=\frac{d_{1i}+d_{2i}}{n_{1i}+n_{2i}}$, and since dead couldn’t reappear we can apply the above “non-replacement” model.

$$E\left(d_{1i}\right)=n_{1i}\frac{d_{i}}{n_{i}}$$

$$D\left(d_{1i}\right)=d_{i}\frac{n_{1i}}{n_{i}}\left(1-\frac{d_{i}}{n_{i}}\right)\frac{n_{i}-n_{1i}}{n_{i}-1}=\frac{n_{1i}n_{2i}d_{i}\left(n_{i}-d_{i}\right)}{n_{i}^{2}\left(n_{i}-1\right)}$$

At time *t_i_ ,* $\frac{\left(d_{1i}-e_{1i}\right)^{2}}{D\left(d_{1i}\right)}$ is distributed $\chi^{2}\left(\alpha, 1\right)$.
